# Efficient production of long double-stranded RNAs applicable to agricultural pest control by *Corynebacterium glutamicum* equipped with coliphage T7-expression system

**DOI:** 10.1007/s00253-021-11324-9

**Published:** 2021-06-07

**Authors:** Shuhei Hashiro, Yasuhiko Chikami, Haruka Kawaguchi, Alexander A. Krylov, Teruyuki Niimi, Hisashi Yasueda

**Affiliations:** 1 Research Institute for Bioscience Products & Fine Chemicals, Ajinomoto Co., Inc., 1-1, Suzuki-cho, Kawasaki-ku, Kawasaki, 210-8681 Japan; 2 Division of Evolutionary Developmental Biology, National Institute for Basic Biology, Nishigonaka 38, Myodaiji, Okazaki, Aichi 444-8585 Japan; 3 Department of Basic Biology, School of Life Science, SOKENDAI (The Graduate University for Advanced Studies), Nishigonaka 38, Myodaiji, Okazaki, Aichi 444-8585 Japan; 4 AGRI (Ajinomoto-Genetika Research Institute), 1-st Dorozhny proezd 1, Moscow, 117545 Russia; 5 Research and Development Center for Precision Medicine, University of Tsukuba, 1-2, Kasuga, Tsukuba-shi, Ibaraki, 305-8550 Japan; 6 Institute for Open Innovation, Kobe University, 1-1, Rokkodai, Nada-ku, Kobe, 657-8501 Japan

**Keywords:** RNA-based pesticide, Double-stranded RNA production, *Corynebacterium glutamicum*, *Henosepilachna vigintioctopunctata*, Colorado potato beetle

## Abstract

**Abstract:**

RNA-based pesticides exert their function by suppressing the expression of an essential gene in the target pest through RNA interference caused by double-stranded RNA (dsRNA). Here, we selected target genes for growth suppression of the solanaceous crop pests ladybird beetle (*Henosepilachna vigintioctopunctata*) and Colorado potato beetle (*Leptinotarsa decemlineata*)-the death-associated inhibitor of apoptosis protein 1 gene (*diap1*), and an orthologous gene of the COPI coatomer protein complex (*copI*), respectively. We constructed a cost-competitive overproduction system for dsRNA using *Corynebacterium glutamicum* as a host bacterium. The dsRNA expression unit was equipped with two sets of promoters and terminators derived from coliphage T7, and the convergent expression system was designed to be selectively transcribed by T7 RNA polymerase. This expression system efficiently overproduced both target dsRNAs. On culture in a jar fermentor, the yield of *diap1*-targeting dsRNA (approximately 360 bp) was > 1 g per liter of culture. Long-chain *diap1*-targeting dsRNAs (up to around 1 kbp) could be produced without a substantial loss of efficiency. dsRNA accumulated in *C. glutamicum* significantly suppressed larval growth of *H. vigintioctopunctata*. The dsRNA expression technology developed here is expected to substantially reduce dsRNA production costs. Our method can be applied for a wide range of industrial uses, including agricultural pest control.

**Key points:**

• *Overexpression of dsRNA was achieved in C. glutamicum using a coliphage T7 system.*

• *The best strain produced > 1 g/L of the target dsRNA species, for use as an insecticide.*

• *The developed system efficiently produced long dsRNA species, up to ~ 1 kbp.*

**Supplementary Information:**

The online version contains supplementary material available at 10.1007/s00253-021-11324-9.

## Introduction

Synthetic chemical pesticides have made a great contribution to supporting the supply of food to mankind. However, conventional chemical pesticides also act indiscriminately on beneficial insects and thus they disrupt ecosystems and adversely affect the natural environment (Woodcock et al. 2016; Hussain et al. 2016). Furthermore, crop pests with acquired resistance to many existing chemical pesticides have emerged because of the extensive/long-term use of these pesticides; this has become a serious problem in recent years (Alyokhin et al. 2007, 2008). Therefore, there is great interest in environmentally friendly pesticides that can replace conventional chemical pesticides. RNA-based pesticides or RNA insecticides, which contain RNA as the essential ingredient, are an example of such technology (Palli 2014; Gu and Knipple 2013).

An RNA-based pesticide is composed of double-stranded RNA (dsRNA). dsRNA can exert an RNA interference (RNAi) effect in the cells of a target pest by acting on mRNA having the same base sequence as the dsRNA. In this way, when a dsRNA molecule created using sequence from an essential gene of a crop pest is incorporated into the pest, expression of the target gene is suppressed and the pest can no longer grow. This effect is not readily applicable to all crop pests, but effective RNAi action has been observed on ingestion of dsRNA in many insects of the order *Coleoptera* (the beetles) (Baum et al. 2007; Palli 2014; Prentice et al. 2017). RNAi effects have also been reported for sanitary pests such as fire ants, termites, and *Tribolium castaneum*, the last of which belongs to the *Coleoptera* (Choi et al. 2012; Zhou et al. 2008; Knorr et al. 2018).

The first challenge in commercializing an RNA-based pesticide is the production cost of the dsRNA. In many studies so far, research on RNAi action on various insects has been carried out using dsRNA prepared via an in vitro transcription system involving T7 RNA polymerase or the similar T7 RNA polymerase/T7 promoter expression system in *Escherichia coli* (Zhu et al. 2011; Yin et al. 2009; Ratzka et al. 2013). However, the former preparation method is not suitable for low-cost dsRNA production, and in the latter, productivity of the target RNAs is too low for useful agricultural application.

In a previous study, we developed a low-cost overproduction system for recombinant RNA using the industrial host microorganism *Corynebacterium glutamicum* (Hashiro et al. 2019b). *C. glutamicum* is a Gram-positive soil bacterium that does not produce endotoxins and is nonpathogenic. This species has been employed as a workhorse for industrial production of many kinds of amino acids for several decades, and has been certified as “generally recognized as safe” (Ikeda and Takeno 2013; Yasueda 2014; Lee and Wendisch 2017). *C. glutamicum* can be stably cultured in a fermentation tank with a capacity of several hundred kiloliters. In the previous investigation, we selected the 28-spotted ladybird beetle *Henosepilachna vigintioctopunctata*, a pest of solanaceous plants such as potatoes, as a model pest, and adopted the essential gene *diap1* (death-associated inhibitor of apoptosis protein 1) as the target of RNAi action (Chikami et al. 2020). dsRNA that can suppress *diap1* gene expression in *H. vigintioctopunctata* (named diap1*-dsRNA, approximately 360-bp long) was successfully overexpressed in *C. glutamicum* (Hashiro et al. 2019c). The strong and constitutive F1 promoter derived from BFK20 (Koptides et al. 1992), a bacteriophage that infects *C. glutamicum*, was used in the study, and a convergent transcription system with F1 promoters was constructed for diap1*-dsRNA production and cloned into a high-copy-number vector (Hashiro et al. 2019a). In a *C. glutamicum* mutant strain lacking *rnc* (encoding RNase III), the dsRNA expression system could accumulate approximately 75 mg of diap1*-dsRNA per liter of culture broth in jar fermentation. The dsRNA produced was shown to cause suppression of *H*. *vigintioctopunctata* feeding activity following oral administration to pest larvae, along with reduced expression of the target gene *diap1* (Hashiro et al. 2019c).

In the present study, we aimed to construct a new dsRNA expression system to further improve the productivity of target dsRNAs. Along with *H*. *vigintioctopunctata*, Colorado potato beetle (CPB; *Leptinotarsa decemlineata*) was selected as a second model pest for this experiment. CPB is a devastating pest of potatoes, especially in North America (Alyokhin et al. 2008; Zhu et al. 2011). The target gene in CPB was an ortholog of *copI*, a gene that encodes a subunit of the coatomer protein complex (COPI), a carrier complex required for retrograde protein transport between the Golgi apparatus and the endoplasmic reticulum (Baum et al. 2014; Baum et al. 2007; Zhu et al. 2011). Previous studies have reported that vital activities in pests including CPB were reduced by the action of RNAi targeting this gene (Baum et al. 2007; Zhu et al. 2011; Kwon et al. 2016; Rodrigues et al. 2017).

In *E. coli*, the T7 expression system derived from coliphage T7 is used extensively for efficient production of proteins of interest. Kortmann et al. (2015) reported an example of using the T7 expression system to produce a target protein in *C. glutamicum*. Previously, we studied use of the T7 system in *C. glutamicum* for unidirectional production of a single-stranded RNA (ssRNA) molecule that partially forms higher-order structure, but the expression level was not markedly superior to that in the system using the F1 promoter (unpublished data). However, the effectiveness of the T7 expression system in convergent transcription for dsRNA production in *C. glutamicum* has not yet been fully evaluated.

Here, we used a *C. glutamicum* strain lacking RNase III (Hashiro et al. 2019b) as the host microbe and constructed a convergent expression system in which two sets of genetic units encoding the T7 promoter and terminator were arranged so as to face each other across the coding region of the target dsRNA. Target dsRNAs were produced very efficiently for both *diap1* and *copI*. This new dsRNA expression system could effectively produce long (~ 1 kbp) dsRNAs. The diap1*-dsRNA-producing strain generated > 1 g of the dsRNA per liter of culture broth in jar fermentation. Taken together, the technology developed here greatly reduces dsRNA production costs and increases the possibility of supplying dsRNA for use in large quantities in agricultural applications.

## Materials and methods

### Bacterial strains, plasmids, and DNA primers

The bacterial strains and plasmids used in this study are listed in Table 1 and Supplemental Table S1. *C. glutamicum* strain 2256LΔ*rnc* (Hashiro et al. 2019b), in which *rnc* (encoding RNase III) is disrupted, was employed as the host strain for dsRNA production. *C. glutamicum* cells were routinely grown on CM-Dex medium (Chinen et al. 2007) in a test tube at 30 °C with reciprocal shaking at 120 rpm, or cultured in a jar fermentor using RPB1 medium (Hashiro et al. 2019b). *E. coli* JM109 (Takara Bio, Shiga, Japan) was used for plasmid construction and the cells were usually cultured in Luria-Bertani medium at 37 °C. When necessary, antibiotics were added as follows: kanamycin (Km) at 25 mg/L and 50 mg/L, and chloramphenicol (Cm) at 5 mg/L and 25 mg/L, for *C. glutamicum* and *E. coli*, respectively. Plasmid pVC7T7pol1, which carries T7 gene *1* (encoding T7 RNA polymerase) expressed under the control of the *lac*UV5 promoter in the backbone of *C. glutamicum*/*E. coli* shuttle vector pVC7N (Hashiro et al. 2019a), was used for inducible production of T7 RNA polymerase in *C. glutamicum*. Isopropyl β-D-1-thiogalactopyranoside (IPTG) was added into *C. glutamicum* culture for induction of transcription from the *lac*UV5 promoter. Plasmid pPK4H1 is a high-copy-number mutant plasmid derived from *C. glutamicum*/*E. coli* shuttle vector pPK4 (Hashiro and Yasueda 2018) and was used as the vector for dsRNA production. pVC7N and pPK4 are compatible with each other in the strain of *C. glutamicum* used in this study. All DNA primers used here are listed in Supplemental Table S2, and all synthetic DNA fragments were obtained from Eurofins Genomics (Tokyo, Japan).

**Table 1:** Bacterial strains and plasmids used in this study

Strain or plasmid	Relevant characteristic(s)^a^	Reference/source
Strain		
*Corynebacterium glutamicum*		
2256	ATCC 13869, AJ1151, wild-type strain	Nishio et al. (2017)
2256L	2256 derivative, cured of cryptic plasmid pAM330N	Hashiro et al. (2019a)
2256LΔ*rnc*	*rnc* mutant of 2256L	Hashiro et al. (2019b)
*Escherichia coli*		
JM109	*endA1*, *recA1*, *gyrA96*,* thi,* *hsdR17* (*r*_k_^−^, *m*_k_^+^),* relA1*, *supE44*, λ^−^, Δ(*lac-proAB*), F′[*traD36*, *proAB*^+^, *lacI*^q^, *lacZ*Δ*M15*]	Takara-Bio (Shiga, Japan)
Plasmid		
pAM330N	Cryptic plasmid in *C. glutamicum* 2256	Hashiro et al. (2019a)
pVC7N	*C. glutamicum–E. coli* shuttle vector derived from pAM330N and pHSG329; Cm^r^	Hashiro et al. (2019a)
pVC54-T7pol	*C. glutamicum–E. coli* shuttle vector carrying T7 gene *1* and *lacI* (*PlacI-lacI-PlacUV5-* T7 gene *1*); Cm^r^	Hashiro et al. (2018)
pVC7T7pol1	pVC7N derivative carrying T7 gene *1* and *lacI *(*PlacI-lacI-PlacUV5*-T7 gene *1*); Cm^r^	K. Haruna (Ajinomoto Co.)
pVC7H2	pVC7N *copA2* mutant; Cm^r^	Hashiro et al. (2019a)
pVH2-HvIap-1	pVC7H2 derivative carrying part (346 bp) of the *diap1*-cDNA and F1 promoters; Cm^r^	Hashiro et al. (2019c)
pHM1519	Cryptic plasmid in *C. glutamicum* ATCC 13058	Hashiro and Yasueda (2018)
pPK4	*C. glutamicum–E. coli* shuttle vector derived from pHM1519 and pHSG299; Km^r^	Hashiro and Yasueda (2018)
pPK4H1	pPK4 *copA1* mutant; Km^r^	Hashiro and Yasueda (2018)
pPK4H1-Pf1-KpnI-XhoI	pPK4H1 derivative carrying *Kpn*I and *Xho*I cleavage sites downstream of F1 promoter; Km^r^	This study
pPH1-HvIap1(F1p)	pPK4H1 derivative carrying part (346 bp) of the *diap1*-cDNA and F1 promoters; Km^r^	This study
pPK4H1-Pt7-KpnI-XhoI	pPK4H1 derivative carrying *Kpn*I and *Xho*I cleavage sites downstream of T7 promoter; Km^r^	This study
pPH1-HvIap1(T7p)	pPK4H1 derivative carrying part (346 bp) of the *diap1*-cDNA and T7 promoters; Km^r^	This study
pPH1-HvIap1(T7p)-200	pPK4H1 derivative carrying part (200 bp) of the *diap1*-cDNA and T7 promoters; Km^r^	This study
pPH1-HvIap1(T7p)-300	pPK4H1 derivative carrying part (300 bp) of the *diap1*-cDNA and T7 promoters; Km^r^	This study
pPH1-HvIap1(T7p)-400	pPK4H1 derivative carrying part (400 bp) of the *diap1*-cDNA and T7 promoters; Km^r^	This study
pPH1-HvIap1(T7p)-500	pPK4H1 derivative carrying part (500 bp) of the *diap1*-cDNA and T7 promoters; Km^r^	This study
pPH1-HvIap1(T7p)-700	pPK4H1 derivative carrying part (700 bp) of the *diap1*-cDNA and T7 promoters; Km^r^	This study
pPH1-HvIap1(T7p)-900	pPK4H1 derivative carrying part (900 bp) of the *diap1*-cDNA and T7 promoters; Km^r^	This study
pPH1-HvIap1(T7p)-1105	pPK4H1 derivative carrying part (1105 bp) of the *diap1*-cDNA and T7 promoters; Km^r^	This study
pPH1-HvIap1(T7p)-1300	pPK4H1 derivative carrying part (1300 bp) of the *diap1*-cDNA and T7 promoters; Km^r^	This study
pPH1-HvIap1(T7p)-1500	pPK4H1 derivative carrying part (1500 bp) of the diap1-cDNA and T7 promoters; Km^r^	This study
pPH1-HvIap1(T7p)-1700	pPK4H1 derivative carrying part (1700 bp) of the *diap1*-cDNA and T7 promoters; Km^r^	This study
pPK4H1-Tt7rev-Pt7-KpnI-XhoI	pPK4H1 derivative carrying T7 terminator and *Kpn*I and *Xho*I cleavage sites downstream of T7 promoter; Km^r^	This study
pPH1-HvIap1(T7pT7t)	pPK4H1 derivative carrying part (346 bp) of the* diap1*-cDNA and T7 promoters and T7 terminators; Km^r^	This study
pPH1-HvIap1L(T7pT7t)	pPK4H1 derivative carrying part (741 bp) of the *diap1*-cDNA and T7 promoters and T7 terminators; Km^r^	This study
pPH1-LdCop1(T7pT7t)	pPK4H1 derivative carrying part (250 bp) of the* copI*-cDNA and T7 promoters and T7 terminators; Km^r^	This study

^a^Cm^r^, resistance to chloramphenicol; Km^r^, resistance to kanamycin

### Insects

Adult *H. vigintioctopunctata* were obtained from the National Institute for Basic Biology (Aichi, Japan), and reared on potato leaves at 25 °C in our laboratory. Larvae used in this study were derived from a few batches of eggs and were starved after second molting, as described previously (Hashiro et al. 2019c).

### Plasmid construction

Plasmid pPK4H1-Pf1-KpnI-XhoI, having cleavage sites for restriction enzymes *Kpn*I and *Xho*I downstream of the F1 promoter, was prepared using a KOD –Plus– mutagenesis kit (Toyobo, Osaka, Japan) as follows. Inverse PCR was performed according to the protocol of the kit using pPK4H1 as the template and primers P01 and P02. After *Dpn*I digestion and self-ligation of the amplified DNA fragment, the product was introduced into *E. coli* JM109 to obtain pPK4H1-Pf1-KpnI-XhoI. Next, PCR was performed using the *diap1*-cDNA fragment (Chikami et al. 2020) as the template and primers P03 and P04, to obtain a *diap1*-cDNA fragment having *Kpn*I and *Xho*I restriction sites at its ends. This fragment was cleaved with *Kpn*I and *Xho*I and ligated to pPK4H1-Pf1-KpnI-XhoI treated with the same restriction enzymes to construct pPH1-HvIap1(F1p).

pPK4H1-Pt7-KpnI-XhoI was constructed by inverse PCR using pPK4H1 as the template and primers P01 and P05. Using the *diap1*-cDNA fragment as the template, a DNA fragment amplified by PCR using primers P03 and P06 was obtained and treated with *Kpn*I and *Xho*I. This fragment was ligated with pPK4H1-Pt7-KpnI-XhoI treated with the same restriction enzymes to generate pPH1-HvIap1(T7p).

A series of 10 fragments of *diap1*-cDNA (200 to 1700 bp long) was obtained by PCR using the cDNA fragment as the template, primer P17, and each of primers P07 to P16 respectively. Each of the 10 amplified DNA fragments was treated with *Kpn*I and *Xho*I, and then ligated with pPK4H1-Pt7-KpnI-XhoI treated with the same restriction enzymes to construct the series pPH1-HvIap1(T7p)-200 to pPH1-HvIap1(T7p)-1700. Another series of 10 diap1*-dsRNA expression plasmids [pPH1-HvIap1(F1p)-200 to pPH1-HvIap1(F1p)-1700] with F1 promoters was constructed in the same manner but using primer P18 instead of primer P17 in the PCR (Supplemental Table S1).

pPK4H1-Tt7rev-Pt7-KpnI-XhoI equipped with a T7 promoter and a T7 terminator was constructed by inverse PCR using the KOD kit, pPK4H1-Pt7-KpnI-XhoI as the template, primers P19 and P20, and self-ligation. Plasmids pPH1-HvIap1(T7pT7t) and pPH1-HvIap1L(T7pT7t) were generated by inserting 346- and 741-bp fragments of *diap1*-cDNA into pPK4H1-Tt7rev-Pt7-KpnI-XhoI, respectively. Each amplified DNA fragment was obtained by PCR using *diap1*-cDNA as the template and primers P23 and P21 or P22. Each was treated with *Kpn*I and *Xho*I, and then ligated with pPK4H1-Tt7rev-Pt7-KpnI-XhoI cleaved with the same restriction enzymes. For PCR and ligation, PrimeStar Max DNA polymerase (Takara Bio) and Ligation high Ver.2 (Toyobo) were employed, respectively.

The expression plasmid pPH1-LdCop1(T7pT7t) for copI*-dsRNA was constructed as follows. A PCR was performed with primers P24 and P25 using *copI*-cDNA as the template to prepare a 250-bp DNA fragment of the cDNA. The DNA fragment was treated with *Kpn*I and *Xho*I, and then ligated with pPK4H1-Pt7-KpnI-XhoI cleaved with the same restriction enzymes to construct pPH1-LdCop1(T7pT7t).

The expression plasmid pVC7T7Pol1 for T7 gene 1 was constructed by transferring the DNA fragment of pVC54-T7pol containing gene 1 into pVC7N, of which we have recently reidentified the full-length DNA sequence (Hashiro et al. 2019a). A DNA fragment obtained by PCR using pVC54-T7pol as the template and primers P26 and P27, and another DNA fragment amplified by PCR with primers P28 and P29 using pVC7N as the template, were linked using an In-Fusion HD Cloning Kit (Takara Bio) to generate pVC7T7pol1.

### Production of dsRNA and polyacrylamide gel electrophoresis (PAGE) analysis

Culture for dsRNA production in a test tube was performed as follows. Transformant colonies were spread on CM-Dex-agar medium containing the necessary antibiotic(s) and cultured overnight at 30 °C. Approximately 0.1 cm^2^ of the bacterial lawn on the plate was inoculated into 2 mL of CM-Dex liquid medium containing antibiotic(s), and the microbial cells were cultured with shaking at 30 °C for 16 h. If addition of IPTG was required, the inducer was added to a final concentration of 1.5 mM 12 h after the start of culture, and the culture was continued for a further 6 h. Total RNA was prepared from 0.2 mL of the culture broth as previously described (Hashiro et al. 2019b), then total RNA solution (1 μL) was mixed with 7 μL of 150 mM NaCl and 2 μL of Hi-Density TBE sample buffer (Thermo Fisher, Tokyo, Japan), and then the sample was analyzed by 6% nondenaturing polyacrylamide gel electrophoresis (PAGE) (Thermo Fisher), followed by staining with SYBR Green II Nucleic Acid Gel Stain (Takara Bio). Batch fermentation was performed in 0.3 L of RPB1 medium containing 120 g/L L-glucose at 30 °C, as described previously (Hashiro et al. 2019b). When production of T7 RNA polymerase was necessary, 2 mM IPTG was added to the culture broth approximately 15 h after the start of the culture, and the fermentation was usually performed for 20–23 h in total. Cell growth was monitored by measuring the optical density of the culture at 620 nm (OD_620_). Quantification of produced RNA was carried out by PAGE analysis using DynaMarker dsRNA (BioDynamics Lab., Tokyo, Japan) as a standard for the calculation (Hashiro et al. 2019b); in the PAGE analysis, the 400-bp dsRNA band in the marker was adjusted to 25 ng per lane. The ratio of segregates in culture broth after fermentation was determined as described previously (Hashiro and Yasueda 2018; Hashiro et al. 2019a). RNase digestion experiments using RNase III (Thermo Fisher) were carried out to characterize the structural features of the produced recombinant RNA, as described previously (Hashiro et al. 2019c). Briefly, total RNA (1.6 μg) extracted from dsRNA-producing cells was digested at 37 °C for 2 h with 500 U of RNaseIII. The digestion reaction was loaded on a 6% acrylamide gel after addition of loading buffer.

### Treatment of *C. glutamicum* cells with ethanol

Cells of *C. glutamicum* for ingestion by insects were prepared by sterilization with ethanol, as described previously (Hashiro et al. 2019c). Briefly, cells were suspended in sodium phosphate buffer (10 mM, pH 6.8) including 80% (v/v) ethanol and the suspension was incubated at 20 °C for 10 min. The microbial cells were collected by centrifugation and then left overnight at room temperature.

### Evaluation of growth inhibition of *H. vigintioctopunctata* larvae

Bioassay using *H. vigintioctopunctata* followed the method described previously (Hashiro et al. 2019c). *C. glutamicum* cells (60 mg wet weight) containing target dsRNA or harboring only vector plasmid (as a control) were sterilized with ethanol as described above and, finally, the samples for feeding were prepared as suspensions in 50 μL of Milli-Q water. Part of the suspension was diluted 1:10 with Milli-Q water. Early third instar larvae of the pest were fed 0.5 μL of diluted or undiluted suspension. After feeding, the larva was transferred onto a fresh potato leaf and raised for 24 h, and then the larva was transferred to new potato leaf on which the rearing was continued for another 24 h. As an evaluation index of the effect of the dsRNA, the larval body weight was measured before and 48 h after feeding the sterilized *C. glutamicum* cells. An analytical balance (AUW120D, Shimadzu, Kyoto, Japan) was employed for body weight measurement. Then, we calculated the increment of the body weight.

Statistical analysis of larval weight was conducted using multiple regression analysis to determine the effect of dsRNA treatment. Multiple regression analysis was conducted using the “lm” function of R-v3.6.0 (R Core Team 2019). After the multiple regression analysis, a multiple comparison analysis for dsRNA treatment was performed by the Tukey method in the “multcomp” package (https://cran.r-project.org/web/packages/multcomp/) (Hothorn et al. 2008).

## Results

### Construction of the dsRNA expression system using the T7 promoter

In a previous study, we constructed a convergent transcription system for dsRNA production using the F1 promoter and high-copy-number vector pVC7H2. *C. glutamicum* carrying the dsRNA expression plasmid (named pVH2-HvIap-1) produced 75 mg diap1*-dsRNA (approximately 360 bp long) per liter of culture broth in a jar fermentor (Hashiro et al. 2019c). The copy number of pVC7H2 was estimated to be around 300 per chromosome in *C. glutamicum* (Hashiro et al. 2019a).

Here, aiming to increase the production level, we adopted vector pPK4H1 (Hashiro and Yasueda 2018) carrying the diap1*-dsRNA expression unit; the copy number of pPK4H1 in *C. glutamicum* is approximately 800 per chromosome. The new expression plasmid was named pPH1-HvIap1(F1p) (Supplemental Fig. S1a). When *C. glutamicum* strain 2256LΔ*rnc* harboring pPH1-HvIap1(F1p) was cultured in a jar fermentor, the dsRNA production reached around 150 mg per liter of culture medium (Supplemental Fig. S1b). No significant difference in the final concentration of cultured *C. glutamicum* cells carrying pVH2-HvIap-1 or pPH1-HvIap1(F1p), or their plasmid retention rates, was observed. Thus, the approximately twofold increase in diap1*-dsRNA accumulation was inferred to mainly be the result of the higher copy number of the expression unit in pPK4H1.

Increasing the number of dsRNA expression units using a higher copy number vector alone did not seem to lead to a dramatic increase in the production of dsRNA, and, therefore, we reconsidered the method of transcribing the convergent expression unit for the target dsRNA using the endogenous RNA polymerase. It has been shown that the RNA polymerase of coliphage T7 is not greatly inhibited in the transcription process in situations where polymerase units collide with each other on opposing DNA strands (Ma and McAllister 2009). Therefore, we tried to apply T7 RNA polymerase to the convergent transcription system in *C. glutamicum*, aiming to further improve the production level of target dsRNAs.

**Fig. 1:**
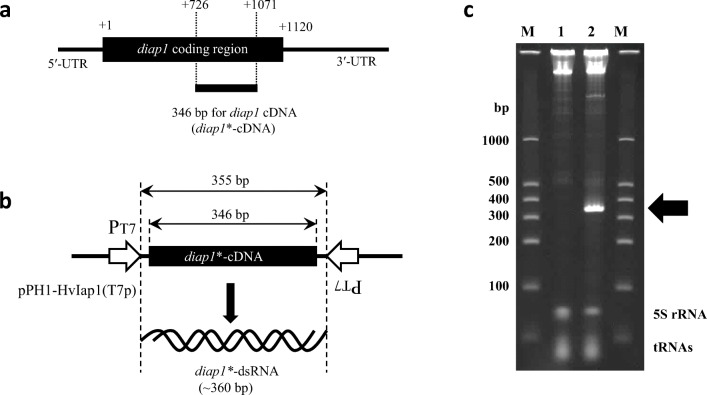
Expression of diap1*-dsRNA by convergent transcription using T7 promoters in *Corynebacterium glutamicum*. **a** Schematic representation of the *Henosepilachna vigintioctopunctata diap1*-cDNA showing the relative position of the diap1*-cDNA. The nucleotide A of the initiation codon ATG is assigned as position +1 of the nucleotide sequence. **b** Structure of *diap*1*-dsRNA expression system in pPH1-HvIap1(T7p). Two T7 promoters (P_T7_) are indicated by open arrows. **c** PAGE analysis of produced diap1*-dsRNA. Lane M shows dsRNA size markers. Total RNA fractions from *C. glutamicum* strain 2256LΔ*rnc* harboring pVC7T7pol1 and pPK4H1 as a control (lane 1), or pVC7T7pol1 and pPH1-HvIap1(T7p) (lane 2). A prominent RNA band corresponding to diap1*-dsRNA is indicated with an arrow, and intrinsic RNAs (5S rRNA and tRNAs) from the host cells are also indicated

As shown in Fig. 1 a and b, therefore, an expression unit was constructed in which two T7 promoters were placed facing each other across the coding region of the target diap1*-dsRNA. This arrangement was cloned into pPK4H1 to construct the expression plasmid, named pPH1-HvIap1(T7p). pVC7T7pol1 was employed to supply T7 RNA polymerase within *C. glutamicum* cells; this plasmid expresses T7 gene *1* (encoding T7 RNA polymerase) under the control of the *lac*UV5 operator/promoter (Supplemental Fig. S2). The backbone pVC7N is compatible with pPK4H1 in *C. glutamicum*. After introducing both plasmids into *C. glutamicum* strain 2256LΔ*rnc*, the dsRNA-producing microbes were cultured in test tubes, and production of T7 RNA polymerase was induced with IPTG. RNAs of the expected length (approximately 360 bp) accumulated to a high level, exceeding the amount of endogenous 5S rRNAs from the host microbe (Fig. 1c). Because the produced RNA band was specifically degraded by RNase III digestion (data not shown) (Hashiro et al. 2019c), it was a dsRNA structure, suggesting that the RNA band was the target diap1*-dsRNA. In a comparison of the level of dsRNA production by pPH1-HvIap1(F1p) and pPH1-HvIap1(T7p) in test tube cultures, the accumulation of the target dsRNA (diap1*-RNA) was about 35 and 46 mg/L, respectively (Fig. 1c, Supplemental Fig. 1b). These data indicated that dsRNA production by the convergent transcription system using the T7 promoter with T7 RNA polymerase was efficiently achieved in *C. glutamicum* 2256LΔ*rnc*. We then proceeded to characterize dsRNA expression by the T7 system, and conducted work to further improve the productivity of dsRNA in *C. glutamicum.*

### Expression of long diap1*-dsRNAs

**Fig. 2:**
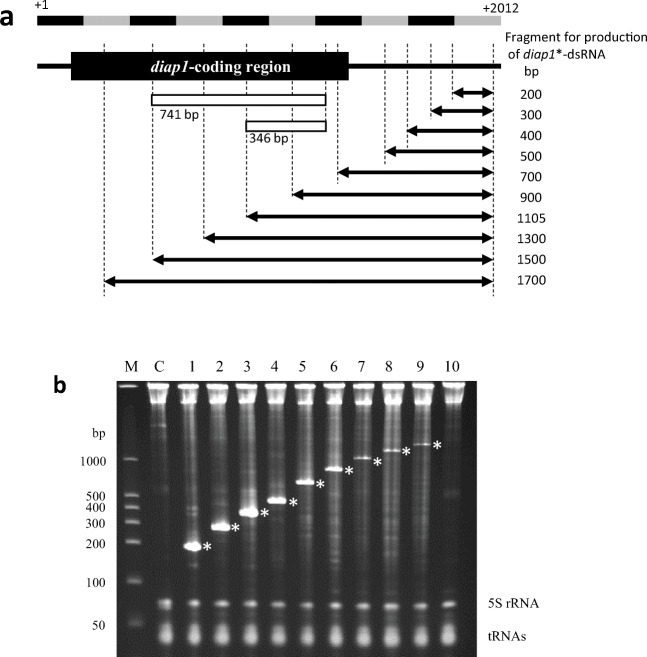
Expression of diap1*-dsRNAs with various nucleotide lengths in *C. glutamicum*. **a** The double-headed arrows under the *diap1*-cDNA indicate the regions of *diap*1*-dsRNA produced using the convergent transcription system. A series of dsRNAs targeting *diap1*-cDNA regions of varying lengths starting from a fixed 3′-end site of the cDNA sequence was expressed in *C. glutamicum* 2256LΔ*rnc*. The open boxes indicate the cDNA regions used for the production of diap1*-dsRNA (~ 360 bp) and diap1*-dsRNA-L (~ 750 bp), respectively. **b** PAGE analysis of total RNA prepared from *C. glutamicum* 2256LΔ*rnc* harboring both pVC7T7pol1 and pPK4H1 (lane C), pPH1-HvIap1(T7p)-200 (lane 1), pPH1-HvIap1(T7p)-300 (lane 2), pPH1-HvIap1(T7p)-400 (lane 3), pPH1-HvIap1(T7p)-500 (lane 4), pPH1-HvIap1(T7p)-700 (lane 5), pPH1-HvIap1(T7p)-900 (lane 6), pPH1-HvIap1(T7p)-1105 (lane 7), pPH1-HvIap1(T7p)-1300 (lane 8), pPH1-HvIap1(T7p)-1500 (lane 9), or pPH1-HvIap1(T7p)-1700 (lane 10). Lane M shows dsRNA size markers. Asterisks on the gel indicate the positions of diap1*-dsRNAs

The length of dsRNA that functions in RNAi is approximately 21–23 bp, but previous dsRNA feeding studies on pests indicated that longer dsRNAs (of a few hundred bp or more) are generally more effective at inducing RNAi effects in insects (Saleh et al. 2006; Kumar et al. 2012; Miller et al. 2012; Khajuria et al. 2015). Thus, we next examined the production of dsRNAs longer than 360 bp. As shown in Fig. 2a, we designed expression vectors to produce a series of dsRNAs of varying lengths (200–1700 bp) starting from a fixed 3′-end site of the cDNA sequence, and then we produced these dsRNAs in *C. glutamicum*. Surprisingly, RNAs of around 1500 bp long were detected as distinguishable RNA bands on PAGE when the total RNA from the producing microbes was separated by electrophoresis (Fig. 2b). In particular, dsRNAs of up to 900 bp long were observed as more intense RNA bands than the intrinsic 5S rRNA band, indicating that target dsRNA production by the T7 expression system in *C. glutamicum* was efficient if the dsRNA was up to around 1 kbp long.

We tried to express the same series of dsRNAs via the convergent transcription system using the F1 promoter instead of the T7 promoter. Target RNA bands of up to approximately 500 bp were clearly observed on PAGE, but the productivity of longer dsRNAs was apparently lower than that by the T7 expression system (Supplemental Fig. S3).

**Fig. 3:**
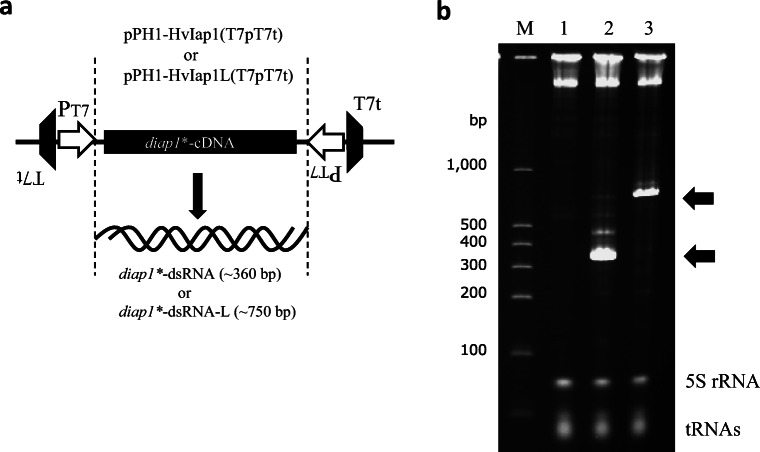
Expression of diap1*-dsRNA using pPH1-HvIap1(T7pT7t). **a** Structure of diap1*-dsRNA expression system using a genetic unit including the T7 promoter (P_T7_) and T7 terminator (T7t). **b** PAGE analysis of produced diap1*-dsRNAs. Lane M shows dsRNA size markers. Total RNA fractions from *C. glutamicum* strain 2256LΔ*rnc* harboring pVC7T7pol1 and pPK4H1 as a control (lane 1), pVC7T7pol1 and pPH1-HvIap1(T7pT7t) (lane 2), or pVC7T7pol1 and pPH1-HvIap1L(T7pT7t) (lane 3). Prominent RNA bands corresponding to diap1*-dsRNA and diap1*-dsRNA-L are indicated with arrows, and intrinsic RNAs (5S rRNA and tRNAs) from the host cells are also indicated. Cells were cultured in test tubes

During experiments into dsRNA expression driven by the T7 system, we often observed RNAs that appeared longer than the expected target dsRNA, appearing as a faint smear in PAGE, when total extracted RNAs were electrophoresed (Figs. 1c and 2b). The appearance of these RNA bands was thought to be because an ssRNA moiety outside the complementary dsRNA-forming region could not be adequately trimmed by RNases in the cell. Thus, we put the T7-derived transcriptional terminator (designated T7t) into the expression system, so that both transcriptions from the strong T7 promoters would terminate at the end of the target dsRNA coding region (Fig. 3a). pPH1-HvIap1(T7pT7t) was constructed, and we examined the productivity of the target dsRNA by the new construct. We could not confirm a convergence in the distribution of the length of the target RNA transcripts by the PAGE analysis. However, we observed significantly increased accumulation of the target dsRNA (Fig. 3b) using the new expression plasmid. Therefore, in subsequent experiments, we used pPH1-HvIap1(T7pT7t) for production of target diap1*-dsRNA of around 360 bp.

The dsRNA production system developed here is applicable to the production of longer RNAs, and therefore we also examined the production of a ~ 750-bp-long diap1*-dsRNA (designated diap1*-dsRNA-L; see Fig. 2a) using the expression plasmid pPH1-HvIap1L(T7pT7t) (Fig. 3a). When the producing microbes were cultured in test tubes and the expression of diap1*-dsRNA-L was induced by adding IPTG, a significant RNA band with the expected length was clearly detected on PAGE (Fig. 3b). This showed again that the convergent expression system using T7 expression units can efficiently express long-chain dsRNA.

### Evaluation of inhibitory effect on *H. vigintioctopunctata* of diap1*-dsRNAs

**Fig. 4:**
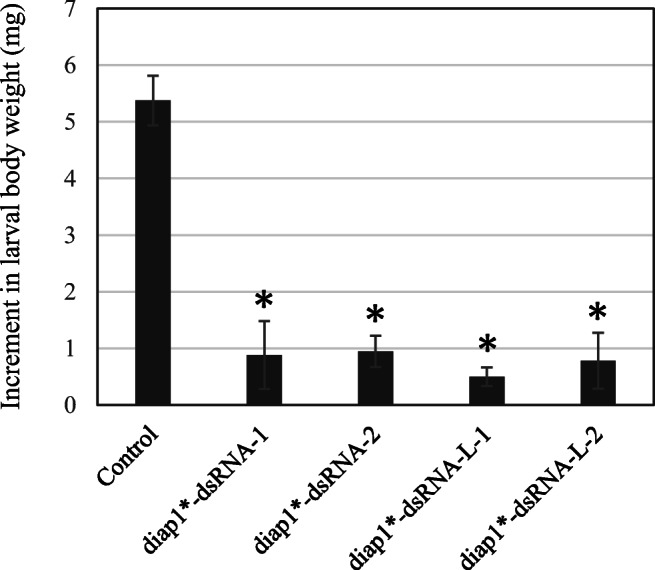
Bioassay of effect of feeding sterilized *C. glutamicum* cells containing diap1*-dsRNAs to *H. vigintioctopunctata* third instar larvae. The difference in larval weight before and 48 h after ingesting the cell suspension is shown. Cells containing the vacant plasmid and pVC7T7pol1 were used as the control. diap1*-dsRNA-1 and -L-1 indicate treatment with undiluted cell suspensions containing diap1*-dsRNA (~ 360 bp) and diap1*-dsRNA-L (~ 750 bp), respectively; diap1*-dsRNA-2 and -L-2 indicate treatment with these cell suspensions diluted tenfold*.* Results are means ± S.D. for seven larvae per group. Statistically significant differences between larvae that ingested the control and those that ingested diap1*-dsRNA samples are marked * (*p* < 0.05). Data on larval weight, coefficients from multiple regression analysis, and results of multiple comparisons using the Tukey method are shown in Supplemental Table S3, Table S4, and Table S5, respectively. It was previously shown that there was no significant difference in the weight gain of larvae between larval groups fed water and those fed sterilized control microbes (Hashiro et al. 2019c)

We previously showed that ethanol-sterilized *C. glutamicum* cells containing diap1*-dsRNA (around 360 bp long, the equivalent of diap1*-dsRNA in this study) reduced the vitality of *H. vigintioctopunctata* larvae (third instar) following oral ingestion (Hashiro et al. 2019c); the larvae showed inhibition of growth associated with reduced potato leaf consumption. Thus, we also examined the effect of the diap1*-dsRNA products generated in the present work on the growth of *H. vigintioctopunctata* larvae. We fed third instar larvae of the pest with sterilized *C. glutamicum* cells containing vacant plasmid vector (pPK4H1, as a control), diap1*-dsRNA, or diap1*-dsRNA-L, and measured the difference in body weight before and 48 h after treatment. During the rearing period, the control larvae gained on average 5.38 ± 0.44 mg (*n* = 7) in body weight, whereas larvae that ingested the cell suspension containing diap1*-dsRNA and diap1*-dsRNA-L gained only 0.88 ± 0.60 mg (*n* = 7) and 0.50 ± 0.17 mg (*n* = 7), respectively. In other words, the body weight increment of larvae treated with the target dsRNA was approximately 9–16% of that of the control larvae (Supplemental Table S3; Fig. 4). By multiple regression analysis, the increase in weight following both types of diap1*-dsRNA treatment was shown to be significantly lower than that in the controls, confirming the biological activity of the dsRNAs (Fig. 4; Supplemental Table S4 and Table S5). Furthermore, we prepared 1:10 diluted cell suspension samples of the original ones and fed them to the pests. The diluted samples had almost the same effect on larval weight gain as the undiluted samples, and no significant differences in the growth inhibition effect were observed between the diap1*-dsRNA and diap1*-dsRNA-L samples (Fig. 4; Supplemental Table S4 and Table S5).

### Overproduction of diap1*-dsRNA in *C. glutamicum* cultured in a fermentor

**Fig. 5:**
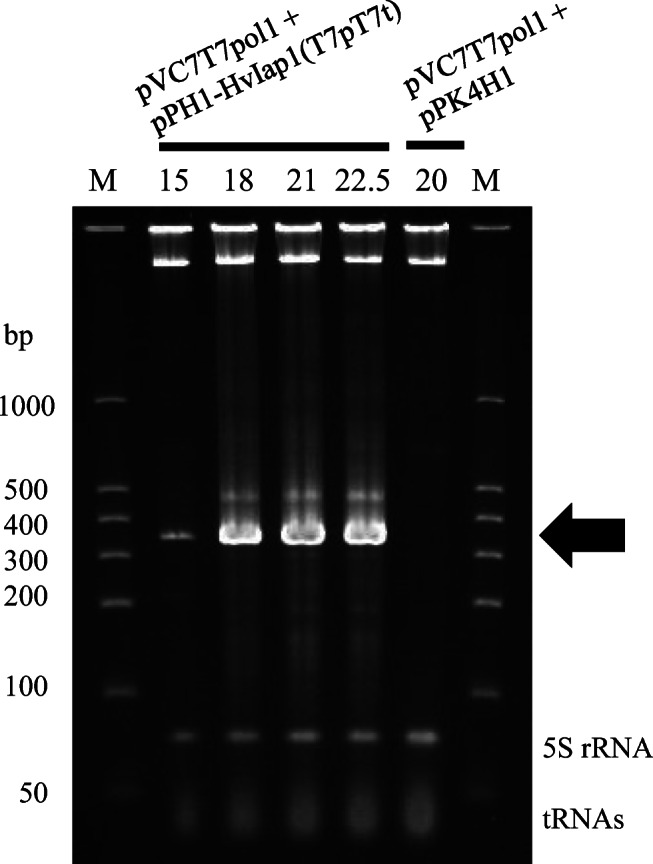
Overproduction of diap1*-dsRNA by *C. glutamicum* in batch fermentation. PAGE analysis of total RNAs prepared from *C. glutamicum* strain 2256LΔ*rnc* harboring pVC7T7pol1 and pPK4H1 (control), or pVC7T7pol1 and pPH1-HvIap1(T7pT7t) during the fermentation. Lane M shows dsRNA size markers, and each total culture time (h) is indicated at the top of the gel. The arrow indicates the position of diap1*-dsRNA. The results show one representative diap1*-dsRNA production experiment in a jar fermentor

To evaluate the maximum yield of the target diap1*-dsRNA that can be produced in *C. glutamicum* using the dsRNA expression system developed here, we performed high-cell-density culture of *C. glutamicum* strain 2256LΔ*rnc* harboring pVC7T7pol1 and pPH1-HvIap1(T7pT7t) in 0.3-L jar fermentor. Fifteen hours after the start of the culture, IPTG was added to induce the expression of T7 gene *1* in the cells, and then the culture was continued until the glucose in the medium was consumed. A maximum OD_620_ value of 160, representing the cell density, was obtained for both the diap1*-dsRNA-producing strain and the control strain. The total RNA extracted from cultured microbes at several points during the cultivation was analyzed by PAGE. As shown in Fig. 5, the target diap1*-dsRNA was prominently detected as the main band at approximately 360 bp on PAGE, although a discrete, faint band at around 470 bp was also observed. After 21 h of total cultivation, the amount of diap1*-dsRNA reached around 1.0 g per liter of culture broth (Supplemental Table S6).

The diap1*-dsRNA-producing microbe contains two compatible plasmids each carrying a different antibiotic resistance marker (Cm or Km), and therefore the resistance of the producer cells to each antibiotic was examined after the culture. Although the T7 RNA polymerase expression plasmid (pVC7T7pol1) was retained fairly stably until the end of the fermentation, pPH1-HvIap1(T7pT7t), carrying the convergent diap1*-dsRNA transcription system, showed a retention rate of ≤ 20%.

### Efficient production of dsRNA targeting the *copI*-orthologous gene of CPB

**Fig. 6:**
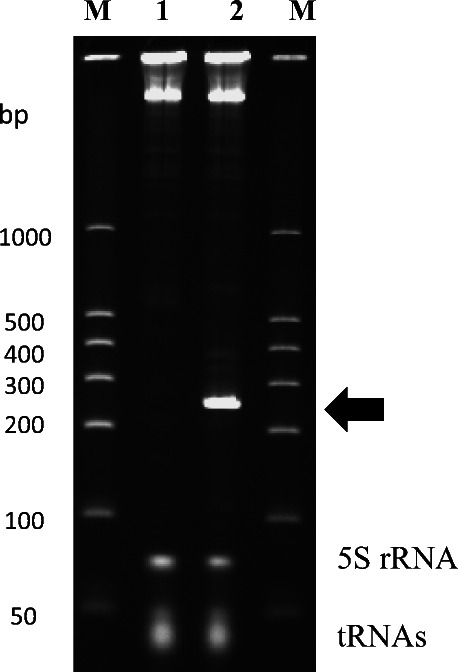
Expression of copI*-dsRNA by *C. glutamicum*. PAGE analysis of total RNAs from *C. glutamicum* 2256LΔ*rnc* harboring pVC7T7pol1 and pPK4H1 as a control (lane 1), or pVC7T7pol1 and pPH1-LdCop1(T7pT7t) (lane 2). Lane M shows dsRNA size markers. A prominent RNA band corresponding to copI*-dsRNA is indicated with an arrow, and intrinsic RNAs (5S rRNA and tRNAs) from the host cells are also indicated

To investigate whether efficient expression of dsRNA that can be applied to other pests was possible using the T7 promoter-dependent system developed in this work, we tried to produce dsRNA targeting the *copI*-orthologous gene of CPB. On the basis of the cDNA sequence of the *copI*-orthologous gene (Supplemental Fig. S4), the expression plasmid pPH1-LdCop1(T7pT7t), in which a 250-bp-long region of *copI*-cDNA was inserted between two T7 promoters (named copI*-dsRNA), was constructed and introduced into *C. glutamicum* 2256LΔ*rnc* cells carrying pVC7T7pol1. When the copI*-dsRNA-producing strain was cultured in test tubes, it was shown that the target dsRNA could be produced with high efficiency (Fig. 6). This result suggested that the dsRNA production system using T7 RNA polymerase and the T7 promoter in *C. glutamicum* is not limited to the *diap1* gene but can be applied to other genes of interest.

## Discussion

The application of dsRNA, instead of chemical pesticides, to crop pest control is highly anticipated because of its expected low impact on the natural environment and its safety for humans. However, the first and biggest challenge is the economical production of dsRNAs. To use dsRNAs as pesticides in the field, it is essential to establish a production system that can supply huge amounts of the target dsRNA. In a previous study on dsRNA production by *C. glutamicum*, we were able to produce a ladybird beetle (*H. vigintioctopunctata*)-derived diap1*-dsRNA at approximately 75 mg per liter of culture via a convergent transcription system using the F1 promoter derived from a corynephage (Hashiro et al. 2019c); however, further improvement of dsRNA production was desired. In this study, when the T7 expression system derived from coliphage T7 was applied to dsRNA production in *C. glutamicum*, we found that highly efficient production could be achieved. On batch culture of the producing microbe, accumulation of a target dsRNA with a chain length of around 360 bp (diap1*-dsRNA) reached approximately 1.0 g per liter of culture. Interestingly, it was also found that the T7 convergent transcription system can efficiently produce dsRNAs with a chain length of up to around 1 kbp (Fig. 2). However, in the system using the F1 promoter and endogenous RNA polymerase in *C. glutamicum*, production of the target dsRNA strongly decreased with increasing chain length (Supplemental Fig. S3). Therefore, it was demonstrated that the expression pattern of dsRNA in the T7 system is significantly different from that in the F1-based system in *C. glutamicum*.

T7 RNA polymerase is relatively small compared with the multisubunit RNA polymerase of *C. glutamicum*, and it has been reported that T7 RNA polymerase can overcome blockage by proteins bound on the DNA strand of a template and perform read-through transcription (Pavco and Steege 1991; Ma and McAllister 2009). This characteristic of T7 RNA polymerase leads us to speculate that the polymerase can avoid collision to some extent in countercurrent transcriptions along the template DNA strands in *C. glutamicum*. This feature may have led to the efficient production of long dsRNAs by the convergent T7 transcription system. Furthermore, because of the high transcription speed of T7 RNA polymerase (Iost et al. 1992), it is speculated that two ssRNAs transcribed by T7 RNA polymerases proceeding in the opposite direction could rapidly form a double-stranded structure between complementary nascent transcripts that then self-fold in the microbe. In such a situation, it is inferred that the dsRNA species generated are not susceptible to attack by ssRNA-degrading RNases within the RNase III-deficient *C. glutamicum* cells and can be accumulated steadily. In fact, in jar fermentation of the dsRNA-producing *C. glutamicum*, even when the dsRNA-producing plasmid [pPH1-HvIap1(T7pT7t)] dropped out of the host cells at a high rate, a large amount of target dsRNA (diap1*-dsRNA) still accumulated in the cells (Fig. 5). This suggests high stability of dsRNA products within the dsRNA-producing *C. glutamicum* strain, although the stability may depend on the specific sequence of the dsRNA. Furthermore, T7 RNA polymerase has higher processivity than multisubunit RNA polymerase, at least the one from *E. coli*. Thus, the insertion of T7t transcription terminators into the dsRNA expression system may also have contributed to the high production of diap1*-dsRNA by pPH1-HvIap1(T7pT7t). The introduction of T7t could have resulted in the pool of T7 RNA polymerase being concentrated in the vicinity of the target dsRNA coding region on the expression plasmid, and thus being devoted exclusively to transcription of that DNA region.

In addition to intrinsic termination, Rho factor-dependent transcriptional termination is known, and *C. glutamicum* has a Rho factor, which is involved in premature termination of transcription by the endogenous RNA polymerase (Takemoto et al. 2015). Thus, in the case of transcription from the F1 promoter by *C. glutamicum* RNA polymerase, some unexpected Rho-dependent transcriptional termination could have occurred. However, it was reported that the Rho factor of *E. coli* does not efficiently terminate transcription by T7 RNA polymerase (Epshtein et al. 2010). In accordance with this finding, it is likely that the Rho factor of *C. glutamicum* also has no significant effect on the activity of T7 RNA polymerase, and thus processive transcription by T7 RNA polymerase may have been achieved in *C. glutamicum* cells, as shown in this study.

The usefulness of the T7 RNA polymerase-dependent gene expression system for overproduction of target proteins in *C. glutamicum* was shown previously (Kortmann et al. 2015), while we have shown for the first time that a dsRNA production system based on convergent transcription using T7 RNA polymerase also works very efficiently in that host species. However, this study also indicated that further improvements are needed to achieve practical dsRNA production. One of the challenges is the induction method for T7 RNA polymerase production. An expensive reagent, IPTG, is required to induce the T7 RNA polymerase expression, which increases the production cost of the target dsRNA. In the *C. glutamicum* strain we used here, strong induction of gene expression from the *lac*UV5 promoter required a slightly higher concentration of IPTG (2 mM) than is usually the case in *E. coli*. This was thought to be due to the low permeability of IPTG into *C. glutamicum* cells, which have a thick cell membrane structure containing mycolic acid (Nishio et al. 2017). To solve this problem, introduction of an uptake system, such as the *E. coli lacY* gene product into the RNA-producing *C. glutamicum*, may be effective (Brabetz et al. 1993). Alternatively, one could use another gene expression system employing a relatively inexpensive inducer, such as arabinose instead of IPTG (Zhang et al. 2012).

A second issue in the practical application of the dsRNA production system developed here is that antibiotic resistance genes are loaded on the plasmids used. Since horizontal gene transfer of drug resistance genes in the environment is a concern in terms of biosafety, it is necessary to take measures to stably maintain the plasmid(s) in the production microbes using alternative genetic markers. For example, use of a gene that complements auxotrophy of the host cell may be appropriate.

Another major issue that needs to be resolved is the in vivo instability of the plasmid carrying the dsRNA transcription system. We observed this especially when production of the T7 RNA polymerase was induced in jar fermentation. In the total RNA extracted from cultured cells, smeared bands of RNA longer than the target dsRNA were observed on PAGE (Fig. 5). These were presumed to be accumulated RNA species of target dsRNA with extra RNA-strand portions derived from read-through transcripts, and we speculated that strong T7 RNA polymerase read-through across the terminator site may be occurring with high frequency. Vilette et al. (1996) showed that simultaneous transcription and replication in opposite directions can frequently promote deletion of DNA in a plasmid with M13 rolling-circle replication. Thus, it is possible that DNA replication of the plasmid was disturbed by high frequency transcriptional intrusion into the plasmid replication control region. A solution to this problem might be the introduction of an additional terminator (Brosius 1984), or replacement with a more efficient terminator (Mairhofer et al. 2015). Furthermore, loading the expression vector with the *par* gene, which serves as a plasmid partition system in coryneform bacteria, may be effective for proper distribution of the target plasmid to daughter cells and stable maintenance of the plasmid (Kurusu et al. 1991). In any case, our experimental results intriguingly showed that the RNA-producing microbes were able to accumulate a large amount of the target dsRNA while losing the dsRNA expression plasmid from the cells. If the expression plasmid can be stabilized, that might be expected to further increase the accumulation of the target dsRNA.

There are some reports that RNA-based pesticides work more effectively when the chain length is a few hundred (or more) base pairs (Saleh et al. 2006; Kumar et al. 2012; Miller et al. 2012; Khajuria et al. 2015). The expression system developed here is suitable for the production of such long-chain dsRNAs. We have not yet conducted a precise and systematic study on the inhibitory effects associated with the different chain lengths of dsRNA on *H. vigintioctopunctata* larvae, but in a bioassay using sterilized microbes containing target dsRNA, we showed that 750-bp-long dsRNA inhibited the growth of the target pest. As our previous study on the evaluation of the growth inhibition of *H. vigintioctopunctata* by diap1*-dsRNA showed that dsRNA feeding reduced the expression of the target gene *diap1* in the pest (Hashiro et al. 2019c), the inhibition of weight gain by both dsRNAs (~ 360 or ~ 750 bp long) in this study is likely due to the RNAi effect of the dsRNA products. In the experiments to evaluate the effect of diap1*-dsRNAs on *H. vigintioctopunctata* in the present work, we did not examine the survival rate of the pest, but we observed a clear inhibition of feeding activity on potato leaves and suppression of larval growth. In our previous study in which in vitro-synthesized *diap1*-dsRNA was fed to larvae orally (Chikami et al. 2020), the treatment caused an acute feeding cessation and death within a few days. Because the essential function of a pesticide is not necessarily to kill the target pest promptly, but rather to inhibit its ability to devour crops, the effectiveness of the dsRNA produced here as a pesticide was demonstrated.

In verifying the toxic effect of RNAi action on a spider mite, Kwon et al. (2016) confirmed that a long-chain chimeric dsRNA linking regions corresponding to two different target genes was more effective against the pest than each dsRNA alone. In addition to *H. vigintioctopunctata*, we examined the productivity of a dsRNA targeting CPB. We showed that cop1*-dsRNA for CPB can also be produced with high efficiency by the T7 gene expression units (Fig. 6), suggesting a degree of versatility in the dsRNA production by the T7 expression system in *C. glutamicum*. Because we have made it possible to produce long dsRNAs with high efficiency here, we can design long-chain dsRNAs in which several dsRNA fragments targeting several species of pest are connected, so that the long dsRNA product could act on multiple target pests. Meanwhile, chimeric long dsRNA species targeting multiple genes of a single pest may inhibit the pest from acquiring resistance to the RNA-based insecticide. Thus, production of such pesticides could be economically attractive.

The effect of the CPB-targeting cop1*-dsRNA on the pest was not tested in this work because of the prohibition on importing CPB into Japan, but the effect of this dsRNA on CPB has already been confirmed in other work (Baum et al. 2014). Therefore, we are convinced that the dsRNA produced here will inhibit the growth of CPB. RNAi through dsRNA can also be applied to sanitary pests such as fire ants (Choi et al. 2012), termites (Zhou et al. 2008), and red flour beetle (Knorr et al. 2018). Furthermore, the application of dsRNA to control viral diseases affecting the shrimp farming industry has recently been studied (Charoonnart et al. 2019; Flegel 2019; Thammasorn et al. 2017). We expect that cost-competitive dsRNAs produced using the production system developed in this study will contribute to various agricultural and aquacultural industries in the near future.

## Supplementary information

ESM 1 (PDF 460 kb)

## Data Availability

The data that support the findings of this study are available from the corresponding authors, H. Y. and S. H., upon reasonable request.
