# Supplementary material for: Efficient production of long double-stranded RNAs applicable to agricultural pest control by Corynebacterium glutamicum equipped with coliphage T7-expression system
Source: Appl Microbiol Biotechnol. 2021 Jun 7;105(12):4987–5000. doi: 10.1007/s00253-021-11324-9 (PMC8236056; doi:10.1007/s00253-021-11324-9)
Supplement: Supplementary file 1 — (PDF 460 kb) [file 253_2021_11324_MOESM1_ESM.pdf]

Applied Microbiology and Biotechnology

Efficient production of long double-stranded RNAs applicable to agricultural pest control by *Corynebacterium glutamicum* equipped with coliphage T7-expression system

Shuhei Hashiro[1,*], Yasuhiko Chikami[2,3], Haruka Kawaguchi[2,3], Alexander A. Krylov[4], Teruyuki Niimi[2,3] and Hisashi Yasueda[1,5,6,*]

[1] Research Institute for Bioscience Products & Fine Chemicals, Ajinomoto Co., Inc., 1-1 Suzuki-cho, Kawasaki-ku, Kawasaki 210-8681, Japan

[2] Division of Evolutionary Developmental Biology, National Institute for Basic Biology, Nishigonaka 38, Myodaiji, Okazaki, Aichi 444-8585, Japan

[3] Department of Basic Biology, School of Life Science, SOKENDAI (The Graduate University for Advanced Studies), Nishigonaka 38, Myodaiji, Okazaki, Aichi 444-8585, Japan

[4] Ajinomoto-Genetika Research Institute (AGRI), 1-st Dorozhny proezd 1, Moscow 117545, Russia

[5] Research and Development Center for Precision Medicine, University of Tsukuba, 1-2 Kasuga, Tsukuba-shi, Ibaraki 305-8550, Japan

[6] Institute for Open Innovation, Kobe University, 1-1 Rokkodai, Nada-ku, Kobe 657-8501, Japan

*Corresponding authors:
Shuhei Hashiro, Email: shuhei_hashiro@ajinomoto.com; Tel: +81-44-210-5892; Fax: +81-44-210-5897
Hisashi Yasueda, Email: yasueda@tiger.kobe-u.ac.jp; Tel: +81-78-803-6512; Fax: +81-78-803-6512

Supplementary Materials

Table S1. Plasmids used in this study

Plasmid	Relevant characteristic(s)[a]	Reference
pPH1-HvIap1(F1p)-200	pPK4H1 derivative carrying part (200 bp) of the *diap1*-cDNA and F1 promoters; Km^r	This study
pPH1-HvIap1(F1p)-300	pPK4H1 derivative carrying part (300 bp) of the *diap1*-cDNA and F1 promoters; Km^r	This study
pPH1-HvIap1(F1p)-400	pPK4H1 derivative carrying part (400 bp) of the *diap1*-cDNA and F1 promoters; Km^r	This study
pPH1-HvIap1(F1p)-500	pPK4H1 derivative carrying part (500 bp) of the *diap1*-cDNA and F1 promoters; Km^r	This study
pPH1-HvIap1(F1p)-700	pPK4H1 derivative carrying part (700 bp) of the *diap1*-cDNA and F1 promoters; Km^r	This study
pPH1-HvIap1(F1p)-900	pPK4H1 derivative carrying part (900 bp) of the *diap1*-cDNA and F1 promoters; Km^r	This study
pPH1-HvIap1(F1p)-1105	pPK4H1 derivative carrying part (1105 bp) of the *diap1*-cDNA and F1 promoters; Km^r	This study
pPH1-HvIap1(F1p)-1300	pPK4H1 derivative carrying part (1300 bp) of the *diap1*-cDNA and F1 promoters; Km^r	This study
pPH1-HvIap1(F1p)-1500	pPK4H1 derivative carrying part (1500 bp) of the *diap1*-cDNA and F1 promoters; Km^r	This study
pPH1-HvIap1(F1p)-1700	pPK4H1 derivative carrying part (1700 bp) of the *diap1*-cDNA and F1 promoters; Km^r	This study

[a] Km^r, resistance to kanamycin.

Table S2. Oligonucleotide DNA primers used in this study

Primer No. (name)	Primer sequence (5'-3')
P01 (KpnI-XhoI_F)	ggtaccaactcgagtacccggggatcctctagagtc
P02 (Pf1_R)	tcgctatctactgtaacatactcatccggtcgcttgaagctggttcgaccgtcccggtaatcatggtcatagctgtttc
P03 (KpnI_F)	aggtaccctcggaatcggcgaccagac
P04 (XhoI-Pf1rev_R)	gctcgagcggtcgaaccagcttcaagttgggggaagagtatgttacagtagatagcgatagaaatacaacagccaattc
P05 (Pt7_R)	ctatagtgagtcgtattagtaatcatggtcatagctgtttc
P06 (XhoI-Pt7rev_R)	gctcgagtaatacgactcactataggtagaaatacaacagccaattc
P07 (KpnI-F_Hv200)	caggtaccgacatttggacgaccattgtatatg
P08 (KpnI-F_Hv300)	caggtacctacgctgtagagagcttcttg
P09 (KpnI-F_Hv400)	caggtaccccgtatgttgcaaactttcttc
P10 (KpnI-F_Hv500)	caggtaccctaaaagtggcagttttggcg
P11 (KpnI-F_Hv700)	caggtaccttgccgtaaagagatccaagc
P12 (KpnI-F_Hv900)	caggtaccgtggtagcaagtagtagcagtag
P13 (KpnI-F_Hv1105)	caggtaccctcggaatcggcgaccagac
P14 (KpnI-F_Hv1300)	caggtaccctctccgttcttgcaaactg
P15 (KpnI-F_Hv1500)	caggtaccttgttgaacgacgatccaatgg
P16 (KpnI-F_Hv1700)	caggtaccctggcgataatggttgttcc
P17 (XhoI-Pt7rev-R_Hv)	cactcgagtaatacgactcactatagggtggaacattacacgatagaac
P18 (XhoI-Pf1rev-R_Hv)	cactcgagcggtcgaaccagcttcaagttgggggaagagtatgttacagtagatagcgtggaacattacacgatagaac
P19 (Tt7rev-insert_F)	agaggccccaaggggttatgctaagaagtttactaatacgactcactatagggtac
P20 (Tt7rev-insert_R)	aaacgggtcttgaggggtttttttgtaatcatggtcatagctgtttc
P21 (KpnI-F_Hv360)	cggtaccctcggaatcggcgaccag
P22 (KpnI-F_Hv741)	cggtaccttgttgaacgacgatccaatgg
P23 (Hv_XhoI-Tt7rev-Pt7_R)	cctcgagaaaaaaacccctcaagacccgtttagaggccccaaggggttatgctacaacttttaatacgactcactataggtagaaatacaacagccaattc
P24 (KpnI-F_CPB250)	cggtaccgtaccgttagagtttggcatacg
P25 (CPB250_XhoI-Tt7rev-Pt7_R)	cctcgagaaaaaaacccctcaagacccgtttagaggccccaaggggttatgctacaacttttaatacgactcactataggccttctggcagcgccttcaaatta
P26 (T7 pol casette_F)	aattcctgtgaattacccgatggtagtgtggggtc
P27 (T7 pol casette_R)	ttagtgatggtgatgttacgcgaacgcgaagtccg
P28 (pVC7_lin_R)	taattcacaggaattggcgtaatcatggtcatagctgtttcc
P29 (pVC7_lin_F)	catcaccatcactaagcttcttccgcttcctcgct

Kpn I and *Xho* I sites in the primer sequence are underlined and double underlined, respectively.

Table S3. Larval weight of *H. vigintioctopunctata* before and 48 h after ingestion of sterilized *C. glutamicum* cells containing *diap1**-dsRNAs[a]

Treatment (dsRNA)	Sample no.	Weight (mg)		Increment (mg)
		Pre-treatment	Post-treatment	
Control	1	1.11	7.36	6.25
	2	1.43	6.46	5.03
	3	1.30	6.14	4.84
	4	1.11	6.51	5.40
	5	1.47	6.62	5.15
	6	1.20	6.47	5.27
	7	1.09	6.79	5.70
	mean			5.38
*diap1**-dsRNA-1	1	1.51	2.18	0.67
	2	1.28	3.54	2.26
	3	1.44	2.23	0.79
	4	1.25	1.82	0.57
	5	1.53	2.12	0.59
	6	1.78	2.05	0.27
	7	1.26	2.30	1.04
	mean			0.88
*diap1**-dsRNA-2	1	1.58	2.06	0.48
	2	2.05	2.95	0.90
	3	1.60	2.85	1.25
	4	1.41	2.61	1.20
	5	1.74	2.94	1.20
	6	1.63	2.28	0.65
	7	1.66	2.62	0.96
	mean			0.95
*diap1**-dsRNA-L-1	1	1.56	1.92	0.36
	2	1.04	1.77	0.73
	3	1.58	1.86	0.28
	4	1.83	2.18	0.35
	5	1.69	2.16	0.47
	6	1.60	2.27	0.67
	7	1.12	1.76	0.64
	mean			0.50
*diap1**-dsRNA-L-2	1	1.45	2.89	1.44
	2	1.14	1.60	0.46
	3	1.29	1.70	0.41
	4	1.72	1.96	0.24
	5	1.77	2.38	0.61
	6	1.54	3.16	1.62
	7	1.27	1.99	0.72
	mean			0.79

[a] *diap1**-dsRNA-1 and -L-1 indicate treatment with undiluted cell suspensions containing *diap1**-dsRNA (~360 bp) and *diap1**-dsRNA-L (~750 bp), respectively; *diap1**-dsRNA-2 and -L-2 indicate treatment with these cell suspensions diluted tenfold.

Table S4. Coefficients from multiple regression analysis of the increment of larval weight of *H. vigintioctopunctata*[a]

	Estimate	SE	*t*-value	*P*-value
(Intercept)	0.0062	0.00048	12.79	1.91E-13
Pre-treatment weight	−0.66	0.37	−1.80	0.082
*diap1**-dsRNA-1	−0.0044	0.00025	−17.72	<2.00E-16
*diap1**-dsRNA-2	−0.0042	0.00028	−14.70	5.65E-15
*diap1**-dsRNA-L-1	−0.0047	0.00025	−18.67	<2.00E-16
*diap1**-dsRNA-L-2	−0.0045	0.00025	−17.93	<2.00E-16

[a] SE, standard error. *diap1**-dsRNA-1 and -L-1 indicate treatment with undiluted cell suspensions containing *diap1**-dsRNA (~360 bp) and *diap1**-dsRNA-L (~750 bp), respectively; *diap1**-dsRNA-2 and -L-2 indicate treatment with these cell suspensions diluted tenfold.
The multiple regression analysis was performed based on the data in Table S3. We used the 'Increment' in Table S3 as an objective variable and the 'Pre-treatment' and 'Treatment (dsRNA)' in Table S3 as explanatory variables. The post-hoc analysis of this result is listed in Table S5.

Table S5. Results of multiple comparison by the Tukey method after multiple regression analysis of the larval weight of *H. vigintioctopunctata*

Samples to be compared		Estimate	SE	*t*-value	*P*-value	Significance[a]
Control	*diap1**-dsRNA-1	−4.37E-03	2.46E-04	−17.72	<1.00E-04	Yes
Control	*diap1**-dsRNA-2	−4.15E-03	2.82E-04	−14.70	<1.00E-04	Yes
Control	*diap1**-dsRNA-L-1	−4.72E-03	2.53E-04	−18.67	<1.00E-04	Yes
Control	*diap1**-dsRNA-L-2	−4.45E-03	2.48E-04	−17.93	<1.00E-04	Yes
*diap1**-dsRNA-1	*diap1**-dsRNA-2	2.17E-04	2.51E-04	0.86	0.91	No
*diap1**-dsRNA-1	*diap1**-dsRNA-L-1	−3.50E-04	2.37E-04	−1.47	0.59	No
*diap1**-dsRNA-1	*diap1**-dsRNA-L-2	−8.64E-05	2.36E-04	−0.37	1.00	No
*diap1**-dsRNA-2	*diap1**-dsRNA-L-1	−5.66E-04	2.45E-04	−2.31	0.17	No
*diap1**-dsRNA-2	*diap1**-dsRNA-L-2	−3.03E-04	2.49E-04	−1.22	0.74	No
*diap1**-dsRNA-L-1	*diap1**-dsRNA-L-2	2.63E-04	2.37E-04	1.11	0.80	No

[a] Significance level is *P*-value < 0.05. SE, standard error. As the control, a suspension of cells containing pPK4H1 and pVC7T7pol1 was employed. *diap1**-dsRNA-1 and -L-1 indicate treatment with undiluted cell suspensions containing *diap1**-dsRNA (~360 bp) and *diap1**-dsRNA-L (~750 bp), respectively; *diap1**-dsRNA-2 and -L-2 indicate treatment with these cell suspensions diluted tenfold.

Table S6. Production amount of *diap1**-dsRNA *in C. glutamicum* in a jar fermentor

**Culture time (h)**	**Total *diap1**-dsRNA (mg/L)[a]**
**15**	**48 ± 13**
**18**	**850 ± 95**
**21**	**1021 ± 78**
**22.5**	**991 ± 158**

[a] Mean value ± standard deviation (*n* = 3).

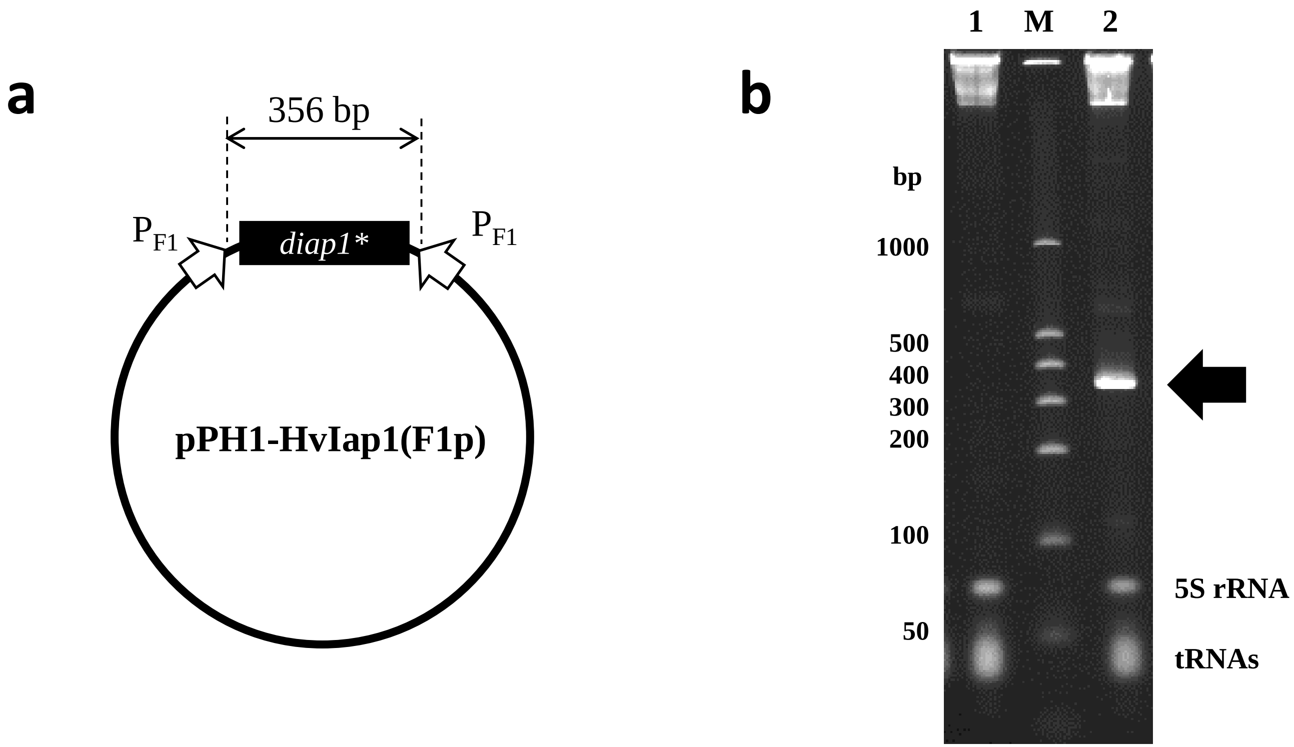

Fig. S1. Production of *diap1**-dsRNA using the F1 promoter. **(a)** A schematic diagram of the structure of expression plasmid pPH1-HvIap1(F1p) for production of *diap1**-dsRNA. *diap1**-dsRNA was designed to be produced in a convergent transcription system using two F1 promoters. The backbone vector is pPK4H1. **(b)** PAGE analysis of *diap1**-dsRNA produced from pPH1-HvIap1(F1p) in *Corynebacterium glutamicum* cultured in test tube. The position of *diap1**-dsRNA is indicated with an arrow. RNAs (5S rRNA and tRNAs) from the host cells are also indicated. Lane M shows size markers of double-stranded RNAs. Lanes 1 and 2 are total RNA fractions from *C. glutamicum* strain 2256LΔ*rnc* harboring pPK4H1 as a control and pPH1-HvIap1(F1p), respectively.

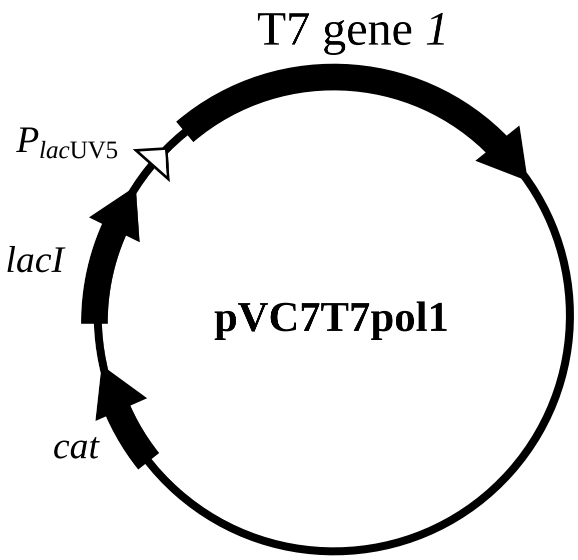

Fig. S2. Schematic diagram showing the structure of expression plasmid pVC7T7pol1 for production of T7 RNA polymerase in *C. glutamicum*. T7 gene *1* was designed to be expressed by transcription from the *lac*UV5 promoter. *lacI* and *cat* genes are also carried on the plasmid. pVC7T7pol1 was a kind gift from Dr. Kenichi Haruna (Ajinomoto Co.).

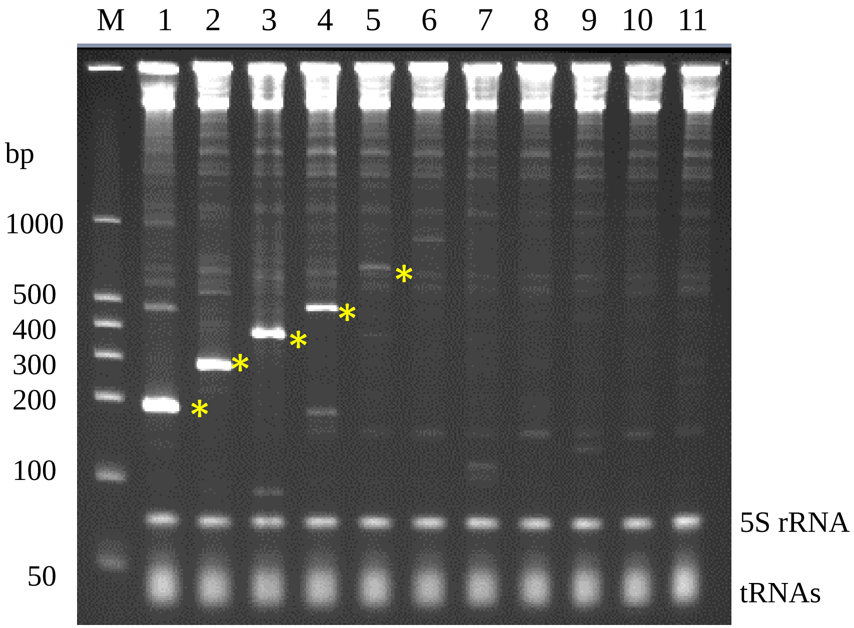

Fig. S3. PAGE analysis of total RNAs prepared from *C. glutamicum* strain 2256LΔ*rnc* harboring pPH1-HvIap1(F1p)-200 (lane 1), pPH1-HvIap1(F1p)-300 (lane 2), pPH1-HvIap1(F1p)-400 (lane 3), pPH1-HvIap1(F1p)-500 (lane 4), pPH1-HvIap1(F1p)-700 (lane 5), pPH1-HvIap1(F1p)-900 (lane 6), pPH1-HvIap1(F1p)-1105 (lane 7), pPH1-HvIap1(F1p)-1300 (lane 8), pPH1-HvIap1(F1p)-1500 (lane 9), pPH1-HvIap1(F1p)-1700 (lane 10), or pPK4H1 (lane 11). Lane M shows dsRNA markers. The yellow asterisks indicate the positions of *diap1**-dsRNAs.

```
5'- CGTAACCGCGGTTTGTTTCCACCCTGAACTACCTGTGGCTCTCACAGGCAGCGAAGATGG :  60
    TACCGTTAGAGTTTGGCATACGAATACACACAGATTAGAGAATTGTTTGAATTATGGGTT : 120
    CGAGAGAGTGTGGACCATTTGTTGCTTGAAGGGTTCGAATAATGTTTCTCTGGGGTATGA : 180
    CGAGGGCAGTATATTAGTGAAAGTTGGAAGAGAAGAACCGGCAGTTAGTATGGATGCCAG : 240
    TGGCGGTAAAATAATTTGGGCAAGGCACTCGGAATTACAACAAGCTAATTTGAAGGCGCT : 300
    GCCAGAAGGTGGAGAAATAAGAGATGGGGAGCGTTTACCTGTCTCTGTAAAAGATATGGG : 360
    AGCATGTGAAATATACCCT -3'                                      : 379
                                                                   nt
```

Fig. S4. DNA sequence coding for putative COPI from *Leptinotarsa decemlineata**.

The DNA region (250 bp) used for production of *copI**-dsRNA is highlighted in yellow.

*Baum JA *et al*., 2016, US9445603B2.
